# Special Issue “Immunomodulatory Molecules in Cancer”

**DOI:** 10.3390/ijms27020728

**Published:** 2026-01-11

**Authors:** Georgios Pantouris

**Affiliations:** Department of Chemistry, University of the Pacific, Stockton, CA 95211, USA; gpantouris@pacific.edu

Cancer is a collection of non-communicable diseases (NCDs) that includes around 200 distinct types [1]. A common feature of all cancers is the uncontrolled division of malignant cells, which can lead to organ failure, infection, or other complications that eventually cause death. Due to the complexity of the tumor microenvironment (TME) [2] and the timing of detection, which very often occurs at an advanced stage [3], cancer is among the leading causes of premature mortality. Over the course of this century, it is projected that cancer may surpass the death rate of cardiovascular diseases (CVDs), becoming the primary cause of death by NCDs worldwide [4]. Of note, in the United States alone, 2,041,910 new cases of cancer and 618,120 cancer-related deaths were projected to occur in 2025 [5].

To disprove these pessimistic forecasts, efforts must focus on early diagnosis and a deeper understanding of cancer at the molecular level. Over the last two decades, biomedical advances [6,7,8,9,10,11] have transformed oncology, shifting cancer treatment from traditional cytotoxic therapies to personalized strategies that target key immunoregulatory molecules in the tumor microenvironment (TME). Supporting these efforts, our Special Issue entitled “Immunomodulatory Molecules in Cancer” aims to highlight such targets and introduce experimental biologics that are currently in the early stage of development.

The eight manuscripts presented herein (Contributions 1–8) are divided into four research articles and four critical reviews, covering diverse types of cancer. Upon reviewing our collection, the reader will learn more about: (i) the types, occurrence, protein partners, and detection methods of CD74-associated oncogenic fusion proteins, (ii) the pathogenic and protective functional roles of the proinflammatory cytokine interleukin-18 (IL-18) in conjunction with the regulatory role of its natural antagonist IL-18 binding protein (IL-18BP), (iii) the double-edged role of the transcription factor interferon regulatory factor 1 (IRF1) in carcinogenesis and immune homeostasis, (iv) the critical role of inflammasomes in cancer as regulators of immune responses, (v) the cancer-promoting properties of the pleotropic enzyme transglutaminase 2 (TG2), (vi) the potential application of serum cereblon (CRBN) levels as a drug response biomarker in multiple myeloma (MM), (vii) the antitumor potencies of chimeric heavy-chain antibodies through blockade of the immune checkpoint molecule sialic acid-binding Ig-like lectin 15 (Siglec-15), and last but not least, (viii) the optimized formulation, dosing, and cryopreservation of the preclinical anti-metastatic vaccine rWTC-MBTA.

Oncogenic fusion proteins, such as the ones described in Vargas et al. (Contribution 1), are cancer-specific molecules formed by the abnormal merging of two distinct genes into a chimeric one. Upon formation and expression, the new protein has uncontrolled activity (e.g., phosphorylation), promoting cancer progression. Because of this property and their cancer-specific characteristics, oncogenic fusion proteins represent highly attractive molecular targets across different types of cancer. After a comprehensive analysis of the CD74-associated oncogenic fusions in cancer patients and tumor samples, Vargas et al. (Contribution 1) demonstrate that these fusions could arise at the earliest stages of lung cancer. Nevertheless, their detection is often pursued after the primary treatment regimen fails and while the tumor has advanced to a late stage. Therefore, this study underscores pitfalls in the routine assessment methods of cancer patients who carry the CD74-associated oncogenic fusions.

Within this issue, the reader will also find discussions of key immunomodulatory molecules that possess a dual functionality in cancer. The comprehensive review by Novick (Contribution 2) emphasizes the context-dependent activities of IL-18/IL-18BP, justifying the importance of regulating this axis in the microenvironment of cancer cells. Aside from describing the key features of IL-18, IL-18 receptor, and IL-18BP, the manuscript breaks down the roles of IL-18 and IL-18BP in health and disease, analyzing their clinical relevance and potential as molecular targets in cancer. Another immunomodulatory molecule equipped with both oncogenic and tumor-suppressing properties is IRF1. In this work, Perevalova et al. (Contribution 3) highlight the central functional role of IRF1 on interferon (INF)-associated cellular events, providing a detailed description of its tumor-suppressive and tumor-promoting functionalities that lead to the regulation of cell cycle, antitumor immune responses, angiogenesis, telomerase activity, and responses to chemo- as well as other types of therapies.

With an emphasis on their cancer-promoting activities, this collection provides insights into inflammasomes and TG2. Inflammasomes are known to contribute to cancer progression by inducing inflammation and tissue damage. The review by Műzes and Sipos (Contribution 4) sheds light on the pathogenic activity of inflammasomes in colorectal cancer, elucidating their functional role in the TME as well as their regulation by epigenetic and autophagy mechanisms. In the case of the pleiotropic enzyme TG2, Yamaguchi et al. (Contribution 5) silenced the TG2 gene in a hepatocellular carcinoma cell line to demonstrate that the enzyme may contribute to cancer cell proliferation, potentially through the PI3K-Akt and Wnt/β-catenin signaling pathways.

The identification of proteins that can serve as prognostic tools in cancer is of significant value for our field, as they can promote effective treatment strategies and estimate post-treatment survival rates. In this context, the study by Gkioka et al. (Contribution 6) explores the potential of CRBN as a predictive and prognostic biomarker in MM patients treated with lenalidomide–dexamethasone (LD). Although the data summarized support the potential of CRBN as a response biomarker, the authors remain cautious due to the influence of the drug regimens combined with lenalidomide.

Beyond highlighting specific immunomodulatory molecules, our collection also introduces two preclinical biologics that are currently in the early stage of development. These agents offer insights into novel strategies that may shape the future of treatment, thereby enriching the scope of our Special Issue. Cheng et al. (Contribution 7) report the generation of three anti-Siglec-15 chimeric antibodies, which are composed of camelid-derived VHH nanobody domains fused to a human IgG Fc. These antibodies showed high binding affinities to human and murine Siglec-15 as well as antitumor activities. Although these experimental therapeutics are still in the early stage of development, the findings by Cheng et al. (Contribution 7) support the potential development of Siglec-15-targeted immunotherapies. In a different study, Ye et al. (Contribution 8) describe the improved formulation of rWTC-MBTA, an experimental vaccine with anti-metastatic potency. While vaccine formulation is a key determinant of efficacy, the findings of this study may guide the development of more effective cancer immunotherapies.

Finally, we would like to thank the authors, peer reviewers, and editorial team whose contributions have made this Special Issue possible. We hope the findings in this collection will inspire the cancer biology community to pursue discoveries that ultimately improve outcomes for cancer patients. Committed to making a continued and meaningful contribution to the field, we are expanding our efforts by creating a related Special Issue entitled “Cancer-Driving Molecules: From Molecular Mechanisms to Novel Therapeutics”. This issue aims to highlight cutting-edge research on the molecular drivers of cancer, from fundamental mechanistic insights to innovative therapeutic strategies, fostering a deeper understanding that can translate into improved clinical outcomes.
